# The walnut genetic resources of INRA: chronological phenotypic data and ontology

**DOI:** 10.1186/s13104-019-4678-1

**Published:** 2019-10-17

**Authors:** Anthony Bernard, Teresa Barreneche, Marine Delmas, Sophie Durand, Cyril Pommier, Fabrice Lheureux, Eloïse Tranchand, Marianne Naudin, Elisabeth Dirlewanger

**Affiliations:** 1 UMR 1332 BFP, INRA, Université de Bordeaux, 33140 Villenave d’Ornon, France; 2 UEA 0393, INRA, 33210 Langon, France; 3 URGI, INRA, Université Paris-Saclay, 78026 Versailles, France; 4 Ctifl, centre opérationnel de Lanxade, 24130 Prigonrieux, France; 5 Station expérimentale de la noix de Creysse, 46600 Creysse, France; 6 Station d’expérimentation nucicole en Rhône-Alpes, 38160 Chatte, France

**Keywords:** Persian walnut, *Juglans regia* L., Phenotyping, Phenology, Ontology, Genetic resources, Germplasm

## Abstract

**Objectives:**

Persian walnut (*Juglans regia* L.), the walnut species cultivated for nut production, is grown worldwide in temperate areas. In this work, chronological phenotypic data have been collected regarding a part of the walnut genetic resources of the French National Institute for Agricultural Research (INRA) of Bordeaux. Using a well described ontology, these data have been collected in order to assess the phenotypic variations among the accessions, and to better manage the germplasm collection. These data can also be helpful for any breeding program as they provide a clear phenotypic characterization of the main cultivars.

**Data description:**

This paper introduces a dataset collected for 150 *J. regia* accessions for a period from 1965 to 2016, and for 3 observation sites, released as comma separated value spreadsheet. It includes observations about phenological traits (e.g. flowering dates), traits related to in-shell walnut (e.g. weight and size), and traits related to kernel (e.g. color). It can be used by other researchers particularly for multi-site phenological studies in the context of climate change since climate data files are also available. In addition, a complete walnut ontology was deposited in this repository and can assist to standardize the management of any walnut germplasm collection.

## Objective

Genetic resources constitute an essential reservoir of allelic diversity for traits currently being used and those not yet used in breeding programs, their management is crucial [1]. In France, Eric Germain and Francis Delort from the Institut National de la Recherche Agronomique (INRA—French National Institute for Agricultural Research) led a walnut breeding program from 1977 to 2005 [2]. The diversity of INRA’s walnut genetic resources with regards to geographical origin is a result of international cooperation established by Eric Germain. He travelled widely and collected diverse plant materials maintained by the *Prunus*/*Juglans* Genetic Resources Center. These materials have been phenotypically characterized for the needs of his breeding program. Nevertheless, it ended in 2007 due to a new orientation for INRA, following a decision to focus on a smaller number of model species, such as *Prunus* and *Malus*.

Until now, these chronological phenotypic data have remained archived. However, they contain valuable information about traits related to phenology, in-shell nut and kernel of walnut cultivars with worldwide origins. Furthermore, a public ontology has been used and adapted during the breeding program. Regarding the context of global competition and climate change, a new French research project called INNOV’noyer started in 2017 in cooperation between the Centre Technique Interprofessionnel des Fruits et Légumes (Ctifl—Fruit and Vegetable Interprofessional Technical Centre) and the INRA Bordeaux-Aquitaine. The objective being to start a new breeding program using marker-assisted selection, and to provide a larger choice of walnut cultivars better adapted to this context. In this way, these data represent a valuable aid for the project and for any researcher who wants to obtain information regarding phenology of a major tree crop species [3]. To our knowledge, these data also provide the most extensive dataset available publicly particularly for the phenology in a walnut germplasm collection, and a complete walnut ontology is reported.

## Data description

Here we present a raw dataset of chronological phenotypic observations of 150 *J. regia* accessions named Ephesis_export (Table 1, Data file 1) publicly available in the “Portail Data INRA” repository. All accessions are grafted trees located in the arboriculture experimental unit in Toulenne (*Prunus*/*Juglans* Genetic Resources Center) near Bordeaux (latitude 44°34′37.442″N–longitude 0°16′51.48″O) and a part of them are also located in the two French walnut experimental stations of Creysse (latitude 44°53′16.529″N–longitude 1°36′23.838″E) and SENuRA (latitude 45°8′38.565″N–longitude 5°17′34.323″E).

**Table 1:** Overview of data files

Label	Name of data file	File types (file extension)	Data repository and identifier (DOI)
Data file 1	Ephesis_export.csv	Comma separated values file (.csv)	Portail Data INRA repository (URGI Plant and Fungi Dataverse, INRA’s Walnut Genetic Resources Observation) (10.15454/58SYJV) [6]
Data file 2	Climate data Creysse from 1992 to 2017.tab	Tabular data file (.tab)	Portail Data INRA repository (URGI Plant and Fungi Dataverse, INRA’s Walnut Genetic Resources Observation) (10.15454/58SYJV)
Data file 3	Climate data SENuRA from 1988 to 2016.tab	Tabular data file (.tab)	Portail Data INRA repository (URGI Plant and Fungi Dataverse, INRA’s Walnut Genetic Resources Observation) (10.15454/58SYJV)
Data file 4	Climate data Toulenne from 1980 to 2011.tab	Tabular data file (.tab)	Portail Data INRA repository (URGI Plant and Fungi Dataverse, INRA’s Walnut Genetic Resources Observation) (10.15454/58SYJV)
Data file 5	WATO-Walnut-Trait-Ontology.csv	Comma separated values file (.csv)	Portail Data INRA repository (LovINRA, Walnut Trait Ontology) (10.15454/AV5RT2) [7]

Toulenne and Creysse locations in southwestern France are both characterized by an oceanic climate and a sandy-silt soil. In contrast, SENuRA location in southeastern France, also with a sandy-silt soil, is in a continental climate. Both Creysse and SENuRA locations belong to the main walnut production areas in France. The 150 *J. regia* accessions represent the maximum genetic diversity of the available collection [4].

This dataset includes mainly observations from Toulenne (50%) and other from Creysse and SENuRA particularly for control cultivars. In the first row, the header shows the accession number (e.g. “RA 0311”), the accession name (e.g. “Franquette”), the trial site (e.g. “Toulenne”), the campaign (e.g. “1991”), and the phenotyped traits (e.g. “BUD_Est_Jd: Date of bud break”). The dataset contains information particularly about traits related to phenology (e.g. Date of bud break, female/male bloom dates), traits related to in-shell walnut (e.g. weight of 100 dry walnuts, shell thickness) and those related to kernel (e.g. weight of 100 kernels, percentage of light colored kernels).

For the range of observation dates, climate data recorded by on-site weather stations are also available in the “Portail Data INRA” repository for each site: Creysse from 1992 to 2017 (Table 1, Data file 2), SENuRA from 1988 to 2016 (Table 1, Data file 3) and Toulenne from 1980 to 2011 (Table 1, Data file 4). These climate data files show daily (except for SENuRA site which gives climate data monthly) the minimum, the mean and the maximum temperature in degrees Celsius. They also include the rainfall level expressed in millimeters.

Phenotype measurements have been performed using a well described ontology based on already existing methodologies [5] and INRA internal protocols. This file named WATO-Walnut-Trait-Ontology (Table 1, Data file 5) is also publicly available in “Portail Data INRA” repository. In the first row, the header shows the variable name and synonyms (e.g. “BUD_Est_Jd”, “Date of bud break”), the reference of the methodologies and the institution (e.g. “Descriptors for walnuts, IPGRI, 1994, 6.1.2”, “INRA”), the trait description (e.g. “when over 50% of terminal buds have enlarged and bud scales have split exposing the green of the leaves inside”) and the scale name and class (“Julian days of the year of evaluation”, “Time”).

Both chronological phenotypic data and ontology could be used by any researcher wishing to obtain phenotypic description of a particular walnut cultivar. It could be also used to conduct a phenotyping campaign using descriptors recognized within the community working on *J. regia*. Finally, it could be useful to assess the effect of climate change on phenology for tree crop species.

## Limitations

Although these observations represent a complete database concerning a large number of accessions which have been phenotyped on more than 30 traits, they contain missing data left blank as many phenotyping data on tree crop species. Those missing data are notably due to particular climatic conditions some years. Then, regarding climate data from the SENuRA observation site, they are not as accurate as the other two because temperature and rainfall data are not given daily but with monthly average. There is also an imbalance in the amount of data for ‘Franquette’ and ‘Lara’ accessions. Indeed, these two cultivars are usually used as controls for experimentation in France. In that respect, more data are available for controls. Finally, the dates of observation (“Campaign” column in Table 1, Data file 1) are heterogeneous. When an accession has been introduced in orchards at the *Prunus*/*Juglans* Genetic Resources Center in year *n*, it has been usually observed in years *n *+ 1, *n *+ 2 and *n *+ 3. However, the year *n* of introduction can vary a lot due to the long process of germplasm establishment.

The “Portail Data INRA” repository is mainly a landing page which hosts the entire dataset. But for more details, readers have the possibility to manage the data with a pleasant interface (e.g. to choose a particular accession) using the “link to data” available on the page. This link brings to the URGI Plant and Fungi platform hosted by the Unité de Recherche Génomique Info (URGI) which is a research unit in genomics and bioinformatics at INRA, dedicated to plants and crop parasites. This platform also provides the passport descriptors such as the country of origin or the pedigree for each accession.

## Data Availability

The phenotypic data described in this Data Note can be freely and openly accessed on the “Portail Data INRA” repository via the identifier INRA’s Walnut Genetic Resources Observation [6] and the following Digital Object Identifier (DOI): 10.15454/58SYJV. The walnut ontology is available on the same repository via the identifier Walnut Trait Ontology [7] and the following DOI: 10.15454/AV5RT2. Please see Table 1 and reference list for details and links to the data.

## Abbreviations

ANRT: Agence Nationale de la Recherche et de la Technologie
Ctifl: Centre Technique Interprofessionnel des Fruits et Légumes
DOI: Digital Object Identifier
INRA: Institut National de la Recherche Agronomique
SENuRA: Station d’Expérimentation Nucicole en Rhône-Alpes
URGI: Unité de Recherche Génomique Info
